# Improving the Accuracy of Metatarsal Osteotomies in Minimally Invasive Foot Surgery Using a Digital Inclinometer: Preliminary Study

**DOI:** 10.3390/s24031022

**Published:** 2024-02-05

**Authors:** Carlos Fernández-Vizcaino, Eduardo Nieto-García, Nadia Fernández-Ehrling, Javier Ferrer-Torregrosa

**Affiliations:** 1 Doctorate School, Catholic University of Valencia San Vicente Mártir, C/Quevedo, 2, 46001 Valencia, Spain; carlos.fernandez@ucv.es; 2 Podiatry Department, Faculty of Medicine and Health Sciences, Catholic University of Valencia San Vicente Mártir, C/Ramiro de Maeztu, 14, 46900 Torrent, Spain; eduardo.nieto@ucv.es (E.N.-G.); nadia.fernandez@ucv.es (N.F.-E.)

**Keywords:** minimally invasive surgery, inclinometer, osteotomy

## Abstract

Minimally invasive foot surgery (MIS) has become a common procedure to treat various pathologies, and accuracy in the angle of metatarsal osteotomies is crucial to ensure optimal results. This randomized controlled trial with 37 patients investigates whether the implementation of a digital inclinometer can improve the accuracy of osteotomies compared to traditional freehand techniques. Patients were randomly allocated to group A (n = 15) receiving inclinometer-assisted surgery or group B (n = 22) receiving conventional surgery. Osteotomies were performed and outcomes were evaluated using an inclinometer. The inclinometer group showed a significant decrease in plantar pressure from 684.1 g/cm^2^ pretreatment to 449.5 g/cm^2^ post-treatment (*p* < 0.001, Cohen’s d = 5.477). The control group decreased from 584.5 g/cm^2^ to 521.5 g/cm^2^ (*p* = 0.001, Cohen’s d = 0.801). The effect size between groups was large (Cohen’s d = −2.572, *p* < 0.001). The findings indicate a significant improvement in accuracy and reduction in outliers when using an inclinometer, suggesting that this technology has the potential to improve surgical practice and patient outcomes in minimally invasive metatarsal osteotomies.

## 1. Introduction

Metatarsal pathology in the foot is a common clinical presentation in medical practice [1]. The surgical approach involves performing osteotomies on the affected metatarsal bones [2,3]. Capital osteotomy, performed by minimally invasive surgery, represents a modern surgical procedure, widely employed to treat a variety of forefoot pathologies [2], offering significant advantages in terms of postoperative recovery [4,5,6] compared to traditional surgical approaches [7].

Nonetheless, it is important to acknowledge that, during these surgical procedures, the variation in the apparent inclination of metatarsal osteotomies can be considerable. Technical errors or intraoperative difficulties [8] can significantly compromise post-operative outcomes [9,10,11]. Since there are no solid evidence-based guidelines to guide the accuracy of these surgical corrections, it has become imperative to minimize the margin of error when performing osteotomies [9,12]. Given this scenario, the use of technology has become a means to optimize surgical practice [13], with an increasing focus on the use of portable devices to improve both surgical technique and patient outcomes, as seen in different surgical pathologies [9].

Inclinometer technology has emerged as an invaluable asset within clinical settings, as evidenced by its demonstrated contributions to enhanced precision and more accurate evaluations in various medical practices [8,14]. Its utilization has notably impacted the realm of surgical procedures, facilitating improved accuracy and meticulousness in intricate operations [12,14]. Specifically, in the context of minimal incision foot surgery, conventional practices often rely on the visual acumen of surgeons coupled with fluoroscopic assessments [15,16,17,18] to execute freehand osteotomies. Nevertheless, a recurrent challenge encountered in these minimal incision surgeries persists: the necessity to perform osteotomies at a specific angle of 45° [18,19,20,21]. This particular requirement poses a considerable hurdle, demanding a high degree of precision and skill from the surgical team. The incorporation of inclinometer technology aims to address this challenge by providing a reliable means to measure and ascertain the accurate angle, thereby potentially revolutionizing the execution of such critical procedures. This technological integration offers the promise of greater procedural accuracy, potentially minimizing errors and optimizing patient outcomes in minimal incision foot surgeries.

The incorporation of such tools into surgical practice could represent a transformative step in enhancing surgical skill acquisition, particularly in the realm of minimally invasive forefoot surgeries. This approach has the potential to reduce the duration of the learning curve [22] significantly, benefiting both surgeons and patients alike by improving procedural efficiency and patient outcomes.

Our principal goal revolves around scrutinizing whether the utilization of this device yields a substantial impact on the angle of inclination during these surgical procedures, and subsequently, whether such implementation leads to discernibly improved outcomes concerning the distribution of plantar pressure in patients. Within this framework, we hold the belief that methodologies integrating a digital inclinometer will showcase enhanced accuracy when juxtaposed with the traditionally employed approach of freehand osteotomies. The anticipation stems from the inherent capabilities of the digital inclinometer to provide precise measurements, offering a potential avenue for heightened surgical precision, thereby potentially mitigating complications and positively influencing patient outcomes in terms of plantar pressure distribution. This study endeavors to elucidate and establish the efficacy of employing a digital inclinometer as a viable and potentially superior alternative in the context of metatarsal osteotomies, paving the way for advancements in surgical techniques and patient care within this domain.

## 2. Materials and Methods

### 2.1. Study Design In Vitro Cadaver Testing

Initially, our study involved an examination of 10 cadaveric anatomical specimens, from which a total of 30 complete metatarsals were procured after conducting metatarsal osteotomies. Among these specimens, 15 osteotomies were carried out on 5 feet by a single surgeon employing the freehand technique, devoid of utilizing an inclinometer. In contrast, the remaining 15 osteotomies were performed on 5 feet by another surgeon utilizing the inclinometer. Both surgeons had access to resin skeletons, providing a standardized environment for the surgical procedures.

Post-surgery, a meticulous radiographic evaluation of all the conducted osteotomies was conducted using an oblique projection at a consistent distance of 50 cm between each specimen. The resultant Dicom files obtained from these radiographs were subjected to thorough analysis and measurement. To ensure precision and accuracy, the evaluation process involved three separate measurements conducted by the same observer utilizing the Osirix software MD (Pixmeno, Switzerland). The mean value derived from these three measurements was then considered for further analysis and comparative assessment.

This methodical approach allowed for a comprehensive assessment of the differences between osteotomies performed with and without the utilization of the inclinometer. By employing radiographic techniques and software-based measurements, we aimed to discern and quantify the potential variations in precision and accuracy associated with these two distinct surgical methodologies. The utilization of standardized radiographic projections and meticulous measurement processes served as pivotal components in ensuring a rigorous and reliable evaluation of the outcomes following the implementation of the inclinometer in metatarsal osteotomies.

### 2.2. Study Design in a Living Patient

We conducted a comparative study involving two distinct groups of patients undergoing identical surgical procedures. The first group, termed “Group With”, received surgical intervention with the assistance of the inclinometer, while the second group, labeled “Group Without”, underwent surgery performed freehand, without the aid of the inclinometer. This trial was meticulously scrutinized and approved by the Research Ethics Committee of the Catholic University of Valencia under the reference code UCV/2020-2021/097, ensuring strict adherence to the ethical principles outlined in the Declaration of Helsinki [23]. Moreover, the trial’s design and the progression of participants through the study were meticulously structured in alignment with the Strengthening the Reporting of Observational Studies in Epidemiology (STROBE) guidelines [24], thereby upholding rigorous standards of study conduct and reporting.

Prior to the commencement of any testing procedures, comprehensive information regarding the study’s objectives, methodologies, potential risks, and benefits was communicated to all participants. Each participant was provided with a detailed written informed consent form, highlighting their voluntary participation and their right to withdraw from the study at any stage without repercussion [23,24]. This critical step ensured that all participants were fully informed about the nature of the study, allowing them to make informed decisions regarding their involvement while upholding the highest standards of ethical conduct and participant autonomy.

### 2.3. Participants

**Figure 1:**
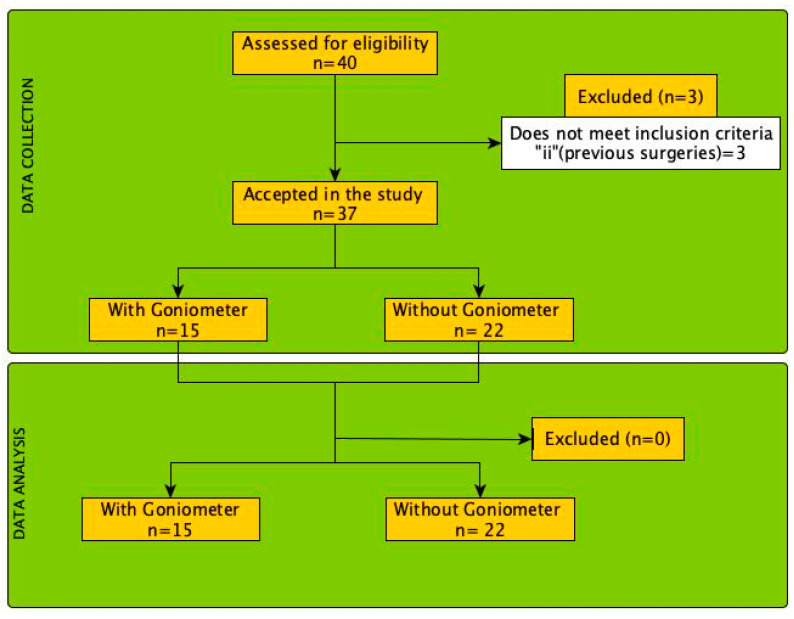
Flow diagram of the selection process and analysis of the patients included in this study.

During the recruitment period spanning from September 2022 to October 2023, a cohort comprising 37 patients afflicted with primary metatarsalgia was systematically enlisted for participation in this study (see Figure 1). The pertinent characteristics of these participants have been meticulously tabulated and are presented in Table 1, providing a comprehensive overview of the measured features crucial to our investigation.

To ensure the selection of suitable candidates, specific eligibility criteria were established for the inclusion of participants in this study. Firstly, individuals were considered eligible if they exhibited persistent metatarsal pain attributed to primary metatarsalgia, thereby aligning with the primary focus of our research. Secondly, the inclusion criteria stipulated those participants had to be over 18 years of age, as this demographic was deemed more representative of the population typically affected by this condition. Lastly, potential participants were required to express their willingness to actively participate in the study and provide informed consent, signifying their acknowledgment and understanding of the study’s objectives, procedures, and any potential risks or benefits associated with their involvement.

These eligibility criteria were crucial in ensuring that the selected participants accurately represented the targeted patient population experiencing primary metatarsalgia. The stringent adherence to these criteria aimed to enhance the homogeneity of the study cohort and uphold ethical standards while ensuring that the data collected would be pertinent and applicable to the specific focus of the investigation.

### 2.4. Surgical Inclinometer

**Figure 2:**
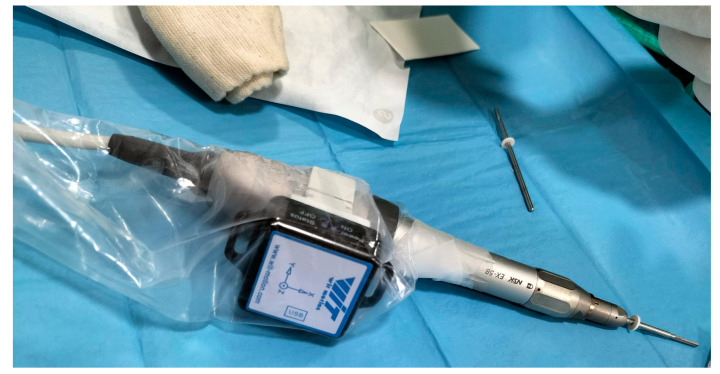
Sensor incorporated in the surgical motor.

The BWT901CL inclinometer (Songgang, China) (see Figure 2) stands as a cutting-edge technological marvel, amalgamating an accelerometer, a 9-axis gyroscope, and a magnetometer to deliver unparalleled accuracy in angle measurement. Its robust design and sophisticated sensors empower it to offer exceptional precision, making it a quintessential tool for diverse applications requiring meticulous tilt measurements. Boasting an impressive accuracy of 0.05 degrees in the XY axes, this inclinometer showcases its prowess by delivering remarkably precise tilt readings, catering to a broad spectrum of applications across various industries and scientific endeavors.

One of its standout features lies in its 3-axis inertial measurement unit (IMU) sensor, operating at an impressive sampling rate of 200 Hz. This attribute ensures steadfast stability and swift responsiveness in detecting orientation changes, providing real-time data essential for making informed decisions during critical procedures. Moreover, the integration of a Kalman filter further bolsters its accuracy by effectively eliminating extraneous noise and unwanted interference. This refinement solidifies its reliability as a formidable instrument, particularly in applications necessitating exceedingly accurate angle measurements, as observed in the intricate osteotomy surgery detailed in our study.

The versatility of the BWT901CL extends beyond its precision and sensor capabilities. Its Bluetooth connectivity feature serves as a gateway, enabling seamless integration with external devices. This functionality facilitates real-time monitoring and data collection, offering a valuable resource for continuous analysis and post-operative follow-up. In our study, this inclinometer was affixed to the surgical handpiece using a specially crafted 3D-printed component tailored to match the motor handle diameter, ensuring a secure and ergonomic attachment during the surgical procedures.

The integration of the BWT901CL inclinometer into the surgical workflow emerges as a testament to its multifaceted utility and adaptability. Its fusion of cutting-edge sensor technology, high precision, noise reduction mechanisms, and connectivity options culminates in a tool that significantly augments the accuracy and efficiency of intricate surgical interventions, exemplifying its pivotal role in advancing medical procedures and enhancing patient care.

### 2.5. Surgical Technique

The surgical technique employed in this procedure necessitates a meticulous approach for precise osteotomy execution. Positioned at a 90-degree angle to the skin surface above the affected metatarsal head, the surgeon aligns parallel to the extensor muscle of the toe. A 2 mm incision is meticulously made using a Beaver 64 scalpel blade, penetrating through the joint capsule to access the underlying bone [21].

The procedure advances to the bone cutting phase utilizing a surgical burr. To accomplish this, a micromotor with RPM control coupled with a high torque reduction handpiece is employed. This choice aims to minimize potential bone damage while conducting the osteotomy, ensuring precision and accuracy in bone restructuring. The specific burr designated for the osteotomy procedure is the Isham Straight Flute Shannon 2.0, chosen based on established literature referencing its suitability for such surgical interventions.

**Figure 3:**
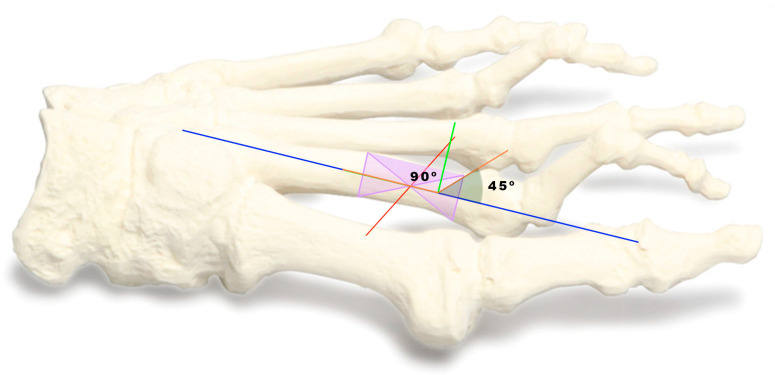
It is placed at a 90-degree angle and the osteotomy is performed at a definitive 45-degree angle. Blue line is the diaphyseal axis of the metatarsal. the green line is perpendicular to the diaphyseal axis (90°) and we position it at 45° degrees to make the metatarsal cut. observing it on the inclinometer.

The angle and direction of the osteotomy are pivotal aspects defining the success of the procedure. The osteotomy angle is set at a definitive 45-degree inclination to the diaphyseal axis of the metatarsal (see Figure 3), as highlighted in various referenced studies [25,26]. The intracapsular burr is guided in a precise direction, commencing the osteotomy from the metatarsal neck’s side, progressing through the plantar cortex, and concluding the cut dorsally. During this process, the burr’s placement remains perpendicular to the metatarsal axis while aligning parallel to the metatarsal’s articular faceta technique emphasized in the existing literature to ensure optimal precision and alignment of the osteotomy [18,21,27].

The detailed execution of each step in this surgical procedure underscores the importance of precision and the adherence to established techniques, as validated by existing scholarly research. These meticulous techniques and procedural intricacies are fundamental to achieving successful metatarsal osteotomies and ultimately contribute to the overall efficacy and positive outcomes in addressing conditions like primary metatarsalgia.

### 2.6. Evaluation Methods

The demographic data encompassing crucial variables such as BMI, the foot subjected to intervention, patient age, and the specific toe targeted for intervention were diligently gathered and are organized within Table 1 for comprehensive analysis. These details were meticulously collated to provide a comprehensive profile of each patient, aiding in the assessment and correlation of various factors with the surgical outcomes.

**Figure 4:**
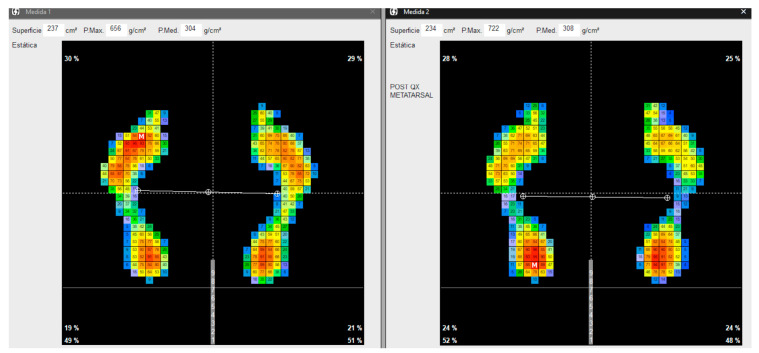
Static pressure measurements.

The assessment of biomechanical aspects and plantar pressure (see Figure 4), crucial components in evaluating foot health, was conducted by an experienced podiatrist (CFV). This assessment involved the utilization of sophisticated equipment to gather precise and reliable data. Specifically, the following tools were employed: a state-of-the-art Medicapteurs S-Plate computerized platform developed by Medicapteurs Co., Balma, France [27,28], a high-performance DELL^®^ computer equipped with robust specifications including a 256 GB SSD, 1.00 TB hard disk, Intel Core^®^ i7 processor, 8.00 GB RAM (Intel, Santa Clara, CA, USA), and running on Windows 10^®^, and a USB 2.0 cable connecting the platform to the computer mainframe.

To ensure accuracy and consistency in the collected data, five recordings were taken from each patient [29]. These multiple recordings aimed to capture a comprehensive range of foot pressures and biomechanical features, providing a robust dataset for analysis. The mean of these measurements was meticulously calculated, a process essential for deriving reliable and representative values for the pressures observed in the treated area.

This rigorous approach to data collection, involving advanced equipment and meticulous recording techniques, facilitated a comprehensive understanding of the biomechanical characteristics and plantar pressure distribution within the cohort of patients under study. Such detailed assessments provide invaluable insights into the specific foot health parameters that may potentially correlate with the outcomes of the surgical intervention, contributing to a more comprehensive evaluation of the efficacy of the surgical procedures employed.

### 2.7. Statistical Analysis

An observer who was unaware of the experimental setup performed all analyses. The mean and standard deviation (SD) were used to express the data. The Kolmogorov–Smirnov test was used to evaluate the assumption of normality. Levene’s test was also used to calculate the homogeneity of variance assumption. At *p* > 0.05, the significance level was established. For the statistical analysis and graphical display of the data, SPSS 24 (SPSS 24 Inc., Chicago, IL, USA) and Jeffreys’s Amazing Statistical Package (JASP V0.16.4, Amsterdam, The Netherlands) were used, respectively. In order to determine whether the anthropometric characteristics among the groups were homogeneous (*p* > 0.05), a one-way *t*-test was used to examine the data. The difference between the groups with and without the inclinometer was analyzed using *t*-test for independent samples (T-Student) and *t*-test for paired samples. In this analysis, the groups (i.e., with and without group) were used as the independent variables. The ES was calculated by determining Cohen’s d coefficient, which was then expressed as the difference of standardized mean change. The ES was categorized as trivial (<0.20), small (0.20–0.59), moderate (0.60–1.19), large (1.20–1.99), or very large (>2.00) [28].

## 3. Outcomes

### 3.1. Outcomes in Cadavers

A statistically significant difference (*p* = 0.030 < 0.05) was observed between the two groups in the osteotomy angulation variable, indicating that the average angulation was significantly different between the groups. The effect size as measured by Cohen’s D was moderate (−0.835), suggesting that the observed difference in means between the groups was of medium–large magnitude. The negative value of Cohen’s D indicates that the group with the inclinometer had a lower mean angulation than the group without (see Table 2)

**Figure 5:**
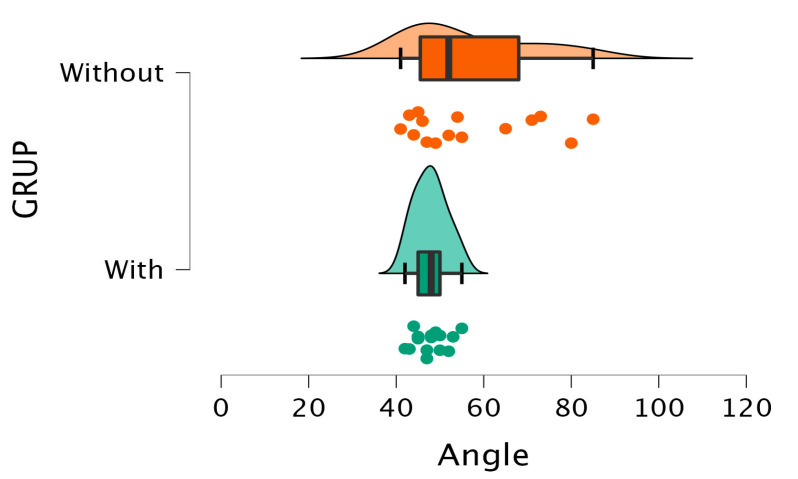
Comparison of scores between the groups when measuring the angle performed in cadaveric osteotomy.

The Brown–Forsythe test was significant (*p* < 0.05), suggesting a violation of the equal variances assumption between groups, which is common in small-sample studies. The mean difference appears to be clinically relevant, since osteotomy angulation is an important variable in these procedures. Visual representations of the angulation distribution in each group (Figure 5) provide further insight into the differences. Additional analyses like normality tests, outlier assessment, homogeneity of variance, etc., could help to understand the dataset behavior.

### 3.2. Outcomes Plantar Pressure

Descriptive analysis of the data (Table 3) shows that in the “Without” group, mean scores decreased from 584.5 (g/cm^2^) before the intervention to 521.5(g/cm^2^) after it (n = 22). In the “With” group, a more significant reduction was observed, from an initial mean of 684.1 to a final mean of 449.5 after treatment (n = 15). The variation coefficients remained moderate in all cases, with values between 0.133 and 0.239.

The comparison of paired pre- and post-measurements using Student’s *t*-test revealed highly significant differences in both the “Without” group (t = 3.756; gl = 21; *p* = 0.001) and the “With” group (t = 21.210; gl = 14; *p* < 0.001). These findings unequivocally demonstrate that the intervention led to statistically significant decreases in the measured scores for both groups.

Moreover, when assessing the effect sizes using Cohen’s d, the observed magnitudes were notably substantial. In the “Without” group, the effect size registered a value of 0.801, classifying as a moderate effect size. Conversely, in the “With” group, the effect size soared to 5.477, indicating an exceptionally large effect size (refer to Table 4).

These effect size measurements, especially in the “With” group utilizing the inclinometer, underscore the substantial impact and significance of the intervention. The remarkable effect size emphasizes the magnitude of change generated by the inclinometer-assisted surgeries, showcasing a robust and notably pronounced impact on the measured scores. This data reinforces the notion that the inclinometer-assisted surgeries resulted in considerably more profound and impactful changes in the measured outcomes compared to surgeries performed without the inclinometer.

**Figure 6:**
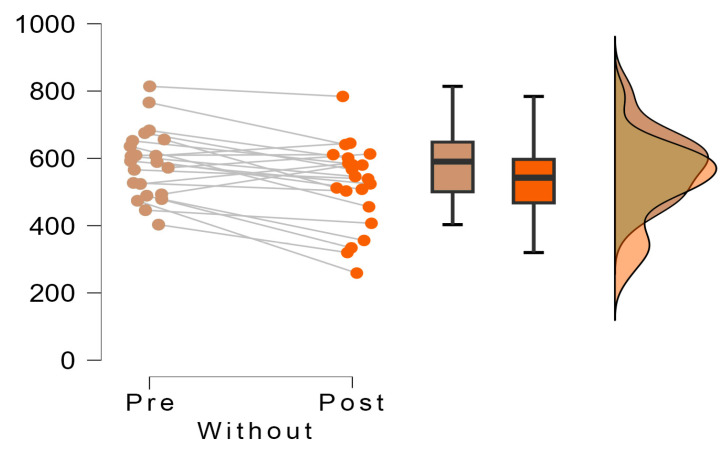
Decrease in pre and post pressures in each study group without the inclinometer.

**Figure 7:**
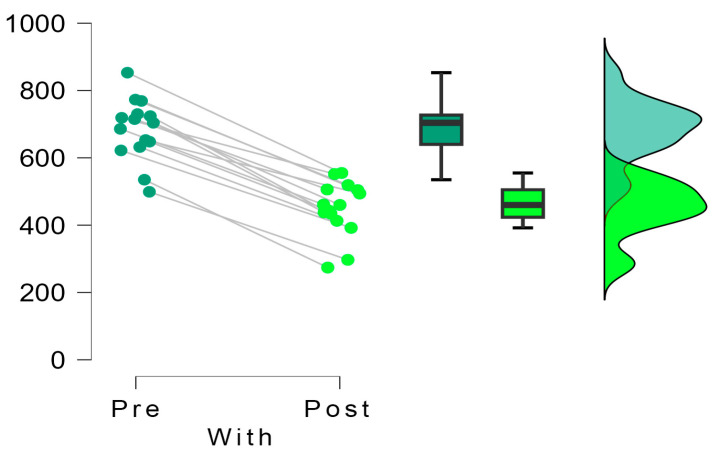
Decrease in pre and post pressures in each study group with the inclinometer.

The insights gleaned from Figure 6 and Figure 7 provide a comprehensive comparison of the dispersion of differences in pre- and post-intervention pressures between the groups that underwent surgery with and without the inclinometer. A notable distinction emerges between the groups: the cohort without the inclinometer displays a considerably higher dispersion in values derived from subtracting the posterior pressure from the initial pressure. This disparity indicates a substantial inter-patient variability in the treatment’s impact within this group.

Conversely, within the group utilizing the inclinometer, a distinct pattern emerges. The pre-post intervention differences for all patients remain closely clustered, consistently positioned on the positive side of the difference axis. This clustering denotes a more uniform and stable scenario, suggesting that patients undergoing surgery facilitated by the inclinometer exhibit consistent decreases in pressure figures post-intervention. Moreover, this trend highlights minimal variation between patients concerning pressure reduction, signifying a more homogenous and consistent response to the intervention.

**Figure 8:**
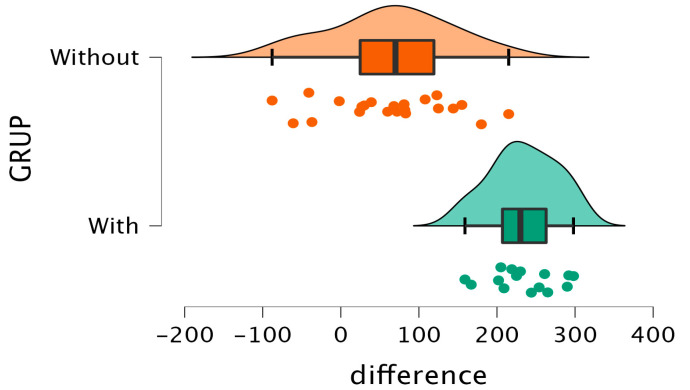
Pressure diagram obtained from the difference between pre- and post-surgery pressures.

The effect size observed between these two groups stands at −2.572 (t = −7.680; gl = 35; *p* < 0.001), underlining the substantial difference in outcomes. This effect size, indicative of the magnitude of the difference between the groups, emphasizes the pronounced impact and statistical significance favoring the utilization of the inclinometer. The data solidly support the fact that the inclinometer-assisted surgeries lead to more consistent and predictable decreases in pressure figures post-intervention compared to surgeries performed without the inclinometer (Figure 8).

## 4. Discussion

Our principal goal revolved around scrutinizing whether the utilization of this device yields a substantial impact on the angle of inclination during these surgical procedures, and subsequently, whether such implementation leads to discernibly improved outcomes concerning the distribution of plantar pressure in patients.

The findings from this study present compelling evidence supporting the notable enhancement in the precision of metatarsal osteotomies through the incorporation of a digital inclinometer within minimally invasive foot surgery, diverging significantly from conventional freehand techniques [18]. This augmented angular accuracy has been prominently showcased in cadaveric specimens, exhibiting a tangible reduction in pressures and a noticeable improvement in the clustering of these diminished pressures when extrapolated to living patients.

The integration of the inclinometer into the surgical motor’s handpiece facilitates a paradigm shift in the surgical landscape, enabling real-time monitoring of the osteotomy’s angulation. This integration parallels the conventional method’s outcomes achieved with standard surgical guides, a practice observed in various fields including podiatry [29,30] and other medical specialties such as dentistry [8].

An instrumental aspect of this technological advancement lies in its ability to reduce reliance on the subjective visual perception and experience of the surgeon [22]. By introducing a standardized technique facilitated by the inclinometer, this approach effectively mitigates inter-operator variability [9].

Initially, our study involved an examination of 10 cadaveric anatomical specimens, from which a total of 30 complete metatarsals were procured after conducting metatarsal osteotomies. The controlled cadaveric environment allowed us to perform direct comparisons between osteotomies conducted with and without the inclinometer in an idealized setting without the constraints of live surgery. This rigorous cadaveric testing enabled precise measurements and evaluations of the angular outcomes, providing critical foundational evidence regarding the potential improvements in accuracy and precision with the inclinometer.

By utilizing this technology, all angles are grouped much closer with reduced variability and standard deviation. In contrast, if we refrain from using the technology and opt for manual measurements, the inclination results tend to be higher and exhibit greater dispersion.

While prior studies have investigated cadaveric foot and ankle surgery to compare techniques [29,31], our study uniquely leveraged cadaveric metatarsal osteotomies to isolate the impact of the inclinometer itself. By standardizing other surgical variables through the cadaver model, we could directly quantify the differences in angular precision between conventional freehand osteotomies and those conducted using the inclinometer. This controlled comparative testing in cadavers paralleled similar research modalities in orthopedic surgery examining techniques [32,33,34,35]. Our cadaveric protocol and testing methods reinforced the validity and potential generalizability of our findings.

Precise angulation holds pivotal significance in the context of this surgical procedure [19], as inaccurately angled osteotomies can profoundly impact the foot’s biomechanics, potentially leading to unfavorable postoperative outcomes [19]. Our research highlights a crucial observation: the utilization of the inclinometer results in a substantial reduction in plantar pressures specifically at the site of the metatarsal head where the osteotomy is performed. This noteworthy improvement in physiological load distribution offers a promising prospect of mitigating complications associated with imperfect angular corrections [31].

Examining the outcomes in patients post-surgery, we note a reduction in plantar pressures observed in both groups due to the nature of metatarsal surgery. However, the magnitude of this effect is notably more pronounced in the group utilizing the inclinometer.

These findings are in alignment with earlier studies that have underscored the utility of inclinometer technology in enhancing accuracy across diverse surgical applications [8,9,12,13,14,36]. Our study builds upon these established findings by reaffirming that the integration of portable angle-measuring devices, such as inclinometers, carries substantial significance, particularly in the domain of minimally invasive foot surgery.

This evidence not only bolsters the case for incorporating inclinometer technology in surgical settings but also emphasizes its potential to revolutionize and significantly improve outcomes in minimally invasive surgical scenarios, specifically in the realm of foot surgery.

This study stands as a significant advancement in the realm of minimally invasive foot surgery by incorporating tilt sensors, offering an intermediate and cost-effective step toward perfecting this field without relying on expensive reference technologies such as the DaVinci system [37,38]. Traditionally, the success of these surgeries heavily relied on the surgeon’s expertise and perception, leading to variability and subjectivity in outcomes [39,40]. However, by seamlessly integrating tilt sensors into foot surgical procedures, this pioneering approach introduces an innovative method that marks a substantial leap forward in minimal incision surgery. This revolutionary methodology not only addresses the limitations associated with the reliance on surgeon expertise but also signifies a noteworthy progression in achieving more precise and standardized surgical outcomes.

It fundamentally alters the conventional landscape by providing a standardized and technologically driven approach to guide these intricate surgeries. By incorporating tilt sensors into the surgical engine, a paradigm shift occurs, enabling the real-time monitoring of angulation throughout osteotomies.

The ability to monitor angulation in real time during osteotomies signifies a monumental leap in surgical practice, promising more standardized and reliable outcomes. This innovation holds immense potential to revolutionize not just foot surgeries but also serves as a beacon for advancements in surgical methodologies across diverse medical specialties.

Moreover, the potential integration of this technology directly into the surgical engine marks a thrilling and progressive development. This prospect allows us to envision a future where surgeons might have seamless access to angle measurement tools integrated directly into their instruments. Such integration could simplify the surgical process further, potentially fostering a more widespread adoption of this innovative technique.

The realm of minimally invasive foot surgery (MIS) is already recognized for its multitude of advantages, including swifter recovery periods [4,5,6] and less scarring [10,17]. The introduction of this innovative technology into MIS procedures holds the promise of further enhancing clinical outcomes for patients undergoing such interventions.

The integration of angle measurement tools directly into surgical instruments not only streamlines the surgical workflow but also presents a significant potential for advancing MIS techniques. This advancement could democratize access to more precise surgical methodologies, potentially benefiting a broader spectrum of patients undergoing foot surgeries.

In its entirety, this study marks a substantial leap forward in the realm of minimally invasive foot surgery (MIS), signaling a promising trajectory toward integrating angle measurement technologies into surgical practice. The successful implementation of inclinometer technology showcased in this study not only advances the precision of MIS foot surgeries but also hints at broader applications across various medical procedures. This innovative integration holds the potential to positively impact surgical accuracy and subsequent clinical outcomes across a spectrum of medical interventions.

While the findings present promising implications, further validation through randomized controlled trials involving larger patient populations is imperative. Confirming the consistent benefits offered by the inclinometer in diverse surgical scenarios will reinforce its reliability and applicability across various medical specialties. Additionally, conducting long-term follow-up studies becomes crucial to ascertain whether the observed improvement in angular accuracy translates into tangible enhancements in clinical and functional outcomes for patients over extended periods.

The necessity for ongoing research underscores the need for rigorous and comprehensive investigation to validate the sustained benefits of inclinometer utilization in surgical settings. These efforts not only validate the initial findings but also shed light on the long-term implications and tangible patient-centered advantages resulting from the integration of such cutting-edge technologies into surgical practice.

The small sample size of just 37 patients and lack of a control group limit the ability to extrapolate the findings to the broader population. Additionally, the narrow focus on metatarsal osteotomies for primary metatarsalgia restricts the generalizability of the results to other areas of foot surgery. The short-term follow-up provides limited data on the durability of outcomes over an extended timeframe. Moreover, the lack of researcher blinding introduces the risk of bias in outcome evaluations. Other unaccounted-for variables like the surgeon’s technique may also influence the results. Ultimately, the observational non-randomized nature of the study means there are uncontrolled confounding factors. More rigorous randomized controlled trials with larger sample sizes and long-term follow-up are required to overcome these limitations and obtain robust conclusions regarding the efficacy of the inclinometer.

A key limitation of this study was its nonrandomized observational design, which means that there are uncontrolled confounding factors that could influence the results. Specifically, variables such as the surgeon’s technique, surgical approach, patient characteristics, and use of postoperative care could differ between groups and introduce bias. To reduce the impact of confounding factors, the studies were performed by the same surgeons with established surgical protocols [11,21,41], and future studies should use randomized controlled trials, strict eligibility criteria, blinding of the outcome assessors, and matched control groups. 

In addition, controlling for factors such as age, weight, comorbidities, and co-interventions through inclusion criteria, statistical adjustments, or subgroup analyses may help to isolate the effect of the inclinometer itself. Larger sample sizes will further minimize the influence of confounding variables.

## 5. Conclusions

This study offers compelling preliminary evidence supporting the efficacy of digital inclinometers in enhancing the precision and consistency of surgical techniques during metatarsal osteotomies performed via MIS surgery. The promising results obtained from this research suggest that the systematic integration of this technology has the potential to optimize both short-term and long-term patient outcomes.

By leveraging digital inclinometers, surgeons can achieve greater accuracy and reproducibility in their surgical procedures, particularly in the context of minimally invasive metatarsal osteotomies. The implementation of these portable devices stands to significantly enhance the precision of these surgical interventions, potentially leading to improved postoperative outcomes for patients.

It is hoped that future trials and ongoing research will delve deeper into exploring the full potential of these portable devices. Continued investigation and trials could further validate and refine the utility of digital inclinometers, setting higher benchmarks for care standards within minimally invasive surgical procedures. The ongoing exploration of these technologies may pave the way for broader adoption, ultimately enhancing the quality of care and bolstering the efficacy of minimally invasive surgical techniques across various medical specialties.

## Figures and Tables

**Table 1:** Descriptions and *t*-tests of general group descriptions per group.

Variable	All Participants (n = 37)	Without (n = 22)	With (n = 15)	*p*-Value
Age	52.324 ± 15.750	51.545 ± 16.795	53.467 ± 14.574	0.721
Height cm	162.8 ± 8.3	162.8 ± 8.5	162.9 ± 8,2	0.986
Weight Kg	66.230 ± 8.95	65.318 ± 8.5	67.56 ± 9.5	0.458
IMC	25.082 ± 3.740	24.729 ± 3.529	25.599 ± 4.099	0.495
Foot Size	38.892 ± 1.941	38.523 ± 1.756	39,433 ± 2.129	0.164
Sides	22 rights 15 lefts	12 rights 10 lefts	10 rights 5 lefts	
Intervened Metatarsal	2° meta (n = 13) 3° meta (n = 19) 4° meta (n = 5)	2° meta (n = 9) 3° meta (n = 10) 4° meta (n = 3)	2° meta (n = 4) 3° meta (n = 9) 4° meta (n = 2)	

**Table 2:** *t*-test for independent samples comparing groups after performing the cadaveric osteotomy.

	t	gl	*p*	Cohen’s D	ET Cohen’s D
Angle (°)	−2.286	28	0.030	^a^ −0.835	0.396

Note. Student’s *t*-test. ^a^ The Brown–Forsythe test is significant (*p* < 0.05), suggesting non-compliance with the assumption of equal variances.

**Table 3:** Mean and standard deviation (SD).

	Pre (g/cm^2^)	Post (g/cm^2^)
Without	584.500 (101.873)	521.455 (124.440)
With	684.133 (90.729)	449.467 (81.924)

**Table 4:** Comparison pre-post (paired samples) and difference in independent sample.

	t	gl	*p*	Cohen’s D	ET Cohen’s D
Without inclinometer pre-post	3.756	21	0.001	0.801	0.164
With inclinometer pre-post	21.210	14	<0.001	5.477	0.502

Note: Student’s *t*-test.

## Data Availability

The data presented in this study are available upon request to the corresponding author. The data are not publicly available due to they are part of a doctoral thesis not yet submitted.
